# Comparative Study of Ozonated Glycerol and Macrogol Ointment on Bone Matrix Production by Human Osteosarcoma Cell Line Saos-2

**DOI:** 10.3390/ma16103857

**Published:** 2023-05-20

**Authors:** Nobutaka Okusa, Hourei Oh, Kazuya Masuno, Yoshimasa Makita, Yasuhiro Imamura

**Affiliations:** 1 Department of Forensic Dentistry, Osaka Dental University, Osaka 573-1121, Japan; 2 Department of Innovation in Dental Education, Osaka Dental University, Osaka 573-1121, Japan; 3 Department of Chemistry, Osaka Dental University, Osaka 573-1121, Japan; 4 Department of Pharmacology, Matsumoto Dental University, Nagano 399-0781, Japan

**Keywords:** ozonated macrogol ointment, ozonated glycerol, viscosity, alkaline phosphatase, collagen, human osteosarcoma cell line Saos-2

## Abstract

Ozonated glycerol is glycerol containing ozone, has no unpleasant odor, and has a long half-life. To apply ozonated glycerol for clinical use, ozonated macrogol ointment has been developed by adding macrogol ointment to ozonated glycerol to increase the retention in the affected area. However, the effects of ozone on this macrogol ointment were unclear. The viscosity of the ozonated macrogol ointment was approximately two times higher than that of ozonated glycerol. The effect of the ozonated macrogol ointment on the human osteosarcoma cell line Saos-2 (Saos-2 cells) proliferation, type 1 collagen production, and alkaline phosphatase (ALP) activity were studied. The proliferation of Saos-2 cells was assessed using MTT and DNA synthesis assays. Type 1 collagen production and ALP activity were studied using ELISA and ALP assays. Cells were treated for 24 h with or without 0.05, 0.5, or 5 ppm ozonated macrogol ointment. The 0.5 ppm ozonated macrogol ointment significantly elevated Saos-2 cell proliferation, type 1 collagen production, and ALP activity. These results also showed almost the same trend as for ozonated glycerol.

## 1. Introduction

Ozone is widely used in society for disinfection, deodorization, disinfecting [1], and clinical use [2,3,4,5,6,7,8]. However, ozone has an unpleasant odor and a short half-life of approximately 40 min [2]. Ozone must be constantly produced to maintain its efficacy. In order to cover this disadvantage, ozonated glycerol (OG) was developed by solubilizing ozone in glycerol in 2004 [9]. Ozonated glycerol is glycerol containing ozone, has no unpleasant odor, and has a long half-life. In fact, ozonated glycerol has been reported to be safe for the skin [10,11], have antimicrobial effects [12], and clarify bone matrix production [13]. Recently, we have shown that ozonated glycerol inactivated SARS-CoV-2 [14].

To apply ozonated glycerol for clinical use, ozonated macrogol ointment has been developed by adding macrogol ointment to ozonated glycerol to increase retention in the affected area. We have shown the anti-inflammatory effect of ozonated macrogol ointment on human gingival fibroblasts and its osteogenic potential on osteoblasts [15]. However, no studies have investigated the effects of macrogol addition in the ozonated gel. The addition of macrogol is essential for clinical use; however, if it alters the properties of the ointment as an ozone gel as demonstrated previously, the macrogol would be unsuitable. Therefore, investigating the effect of adding macrogol is crucial, and addressing this issue is necessary to ensure the efficacy of the ointment as an ozone gel for clinical use. In this study, to evaluate the effect of macrogol addition in ozonated gel, we selected the Saos-2 cells [13,16] and compared the ozonated glycerol and macrogol ointment. The effect of ozonated macrogol ointment on type 1 collagen production and ALP activity in Saos-2 cells was investigated.

## 2. Materials and Methods

### 2.1. Ozonated Macrogol Ointment

The ozonated macrogol ointment was provided by Mediplus Pharma Inc., Tokyo, Japan. The preparation procedure of the ozonated macrogol ointment is below. Ozone gas was administered to concentrated glycerol (Sakamoto Yakuhin Kogyo Co., Ltd., Tokyo, Japan) to produce 2000 ppm of ozonated glycerol using an Ozonizer (Mediplus Pharma Inc., Tokyo, Japan). In addition, a macrogol ointment (polyethylene glycol (PEG) 400 and PEG 4000: Yoshida Pharmaceutical Co., Ltd., Tokyo, Japan) and a water-soluble ointment (polyethylene glycol) were added to increase the viscosity of ozonated glycerol for the purpose of retention. The ratio of macrogol ointment to ozonated glycerol was 1:2 [15]. The viscosity of ozonated glycerol ointment is 30,000 mPa·s. Ozonated macrogol ointment is miscible with water (24.4 g/100 mL), and miscible with ethanol, acetone, and benzene. The degradation time of ozonated macrogol ointment is about three months at room temperature.

### 2.2. Viscosity Measurement

The shear rate in the B-type viscometer BM II (Toki Sangyo Co., Ltd., Tokyo, Japan) was calculated by measuring in advance a Newtonian fluid of known viscosity (viscosity standard fluid) at different rotor speeds [17].

### 2.3. DNA Synthesis and MTT Assays

For DNA synthesis, Saos-2 cells (1 × 10^4^) were cultured in DMEM containing 0.5% FBS (0.5% DMEM) for 24 h. Cells were cultured with 0.05, 0.5, or 5 ppm ozonated macrogol ointment for 2 min. The ozone concentration is the final concentration for each well. The culture medium was then removed. The cells were washed with 0.5% DMEM and cultured with 0.5% DMEM containing bromodeoxyuridine (BrdU) for 24 h. The level of DNA synthesis was determined by measuring BrdU incorporation using a BrdU cell proliferation assay kit (Millipore, Tokyo, Japan). For the MTT (3-(4,5-dimethylthiazol-2-yl)-2,5-diphenyl tetrazolium bromide; Sigma-Aldrich, St. Louis, MO, USA) assay, cells were cultured with ozonated macrogol ointment at 0.05, 0.5, or 5 ppm in DMEM containing 10% FBS (10% DMEM) for 2 min, and the culture medium was then removed. After washing with 10% DMEM, cells were cultured with 10% DMEM for 24 h. The subsequent procedures were performed as previously described [18]. The microplate used a 96-well plate, catalog No. 655 180, manufactured by Greiner. Three wells were used for one sample to be analyzed. The analysis was performed three times for the same experiment.

### 2.4. Enzyme-Linked Immunosorbent Assay (ELISA)

For collagen production, Saos-2 cells (1 × 10^4^) were cultured with 0.5 ppm ozonated macrogol ointment in DMEM containing 1% FBS (1% DMEM) for 2 min. As a control, Saos-2 cells were cultured without ozonated macrogol ointment. Cells were washed and cultured with 1% DMEM. Levels of type 1 collagen in the media were measured by indirect ELISA using the biotinylated anti-type 1 collagen antibody (0.2 µg/mL; Rockland Immunochemicals, Inc., Limerick, PA, USA) and samples (360 µg/mL protein). ELISA was performed as described in the user manual of a CytoSet kit (Biosource, Tokyo, Japan) [19]. Cells for collagen production were lysed with 0.5% Triton X-100, and the protein concentration of the cell lysates was measured using a bicinchoninic acid (BCA) protein assay kit (Pierce, Tokyo, Japan). Collagen production was normalized to the protein content of the cell lysates. The microplate was used on a 96-well plate. Three wells were used for one sample to be analyzed. Samples were measured with a Microplate Reader model 550 (Bio-Rad Labs, Inc., Hercules, CA, USA) using a dual wavelength of 450/655 nm (test/reference). The analysis was performed three times for the same experiment.

### 2.5. ALP Activity

Saos-2 cells (1 × 10^4^) were cultured with a 0.5 ppm ozone macrogol ointment for 2 min. The culture medium was then removed. Cells were washed with 0.5% DMEM and cultured with 10% DMEM for 24 h. Cells were lysed with 0.05% Triton X-100, and the ALP activity of the lysates was measured using a LabAssay ALP kit (Wako Pure Chemicals Industries, Ltd., Osaka, Japan). Protein concentrations of cell lysates were also measured using a BCA protein assay kit. ALP activity was normalized to the protein content of the cell lysates. The microplate was used on a 96-well plate. Three wells were used for one sample to be analyzed. Samples were measured by the Microplate Reader model 550 using a wavelength of 405 nm. The analysis was performed three times for the same experiment.

### 2.6. Statistical Analysis

Quantitative data were statistically analyzed using either a one-way analysis of variance (ANOVA) followed by Tukey’s test (DNA synthesis and MTT assays) or the Student’s *t*-test (measurement of ALP activity and ELISA for collagen production) using the StatMate software (ATMS Co., Ltd., Tokyo, Japan). Differences were considered significant at *p* < 0.05.

## 3. Results

### 3.1. Viscosity Measurement of Ozonated Glycerol and Macrogol Ointment

The viscosity of the ozonated glycerol and the ozonated macrogol ointment were 1499 and 3000 mPa·s, respectively. The viscosity of the ozonated macrogol ointment was about two times greater than that of the ozonated glycerol.

### 3.2. Effect of Ozonated Macrogol Ointment on the Proliferation of Saos-2 Cells

**Figure 1:**
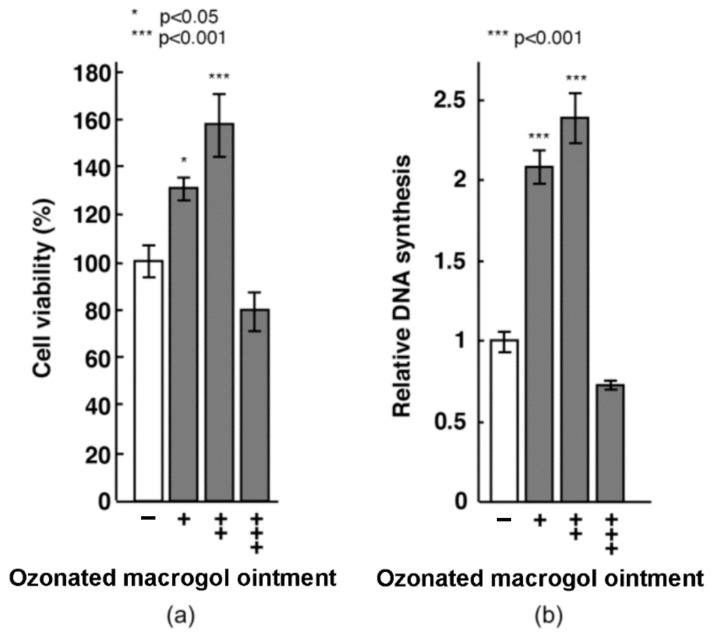
Effects of ozonated macrogol ointment on cell survival and DNA synthesis in Saos-2 cells. (**a**) MTT assay and (**b**) DNA synthesis assay. Saos-2 cells (1 × 10^4^) were seeded into the 96-well plate and exposed to the medium containing 0.05, 0.5, or 5 ppm ozonated macrogol ointment (designated as +, ++, and +++, respectively) for 24 h. All data were compared to that of the control (without ozonated macrogol ointment). Data are presented as mean ± standard deviation (*n* = 3). * *p* < 0.05 and *** *p* < 0.001 vs. control, analyzed with a one-way analysis of variance and Tukey’s test.

After 24 h of incubation, the ozonated macrogol ointment treatment increased Saos-2 proliferation in a dose-dependent manner (Figure 1). Ozonated macrogol ointment at 0.5 ppm significantly induced cell growth compared to that at other concentrations, whereas no obvious change was observed with ozonated macrogol ointment at 5.0 ppm. In line with the results of the MTT assay, 0.5 ppm ozonated macrogol ointment effectively elevated DNA synthesis in Saos-2 cells, indicating 0.5 ppm as the optimal concentration of ozonated macrogol ointment required for facilitating cell proliferation.

### 3.3. Effect of Ozonated Macrogol Ointment on Type 1 Collagen Production and ALP Activity by Saos-2 Cells

**Figure 2:**
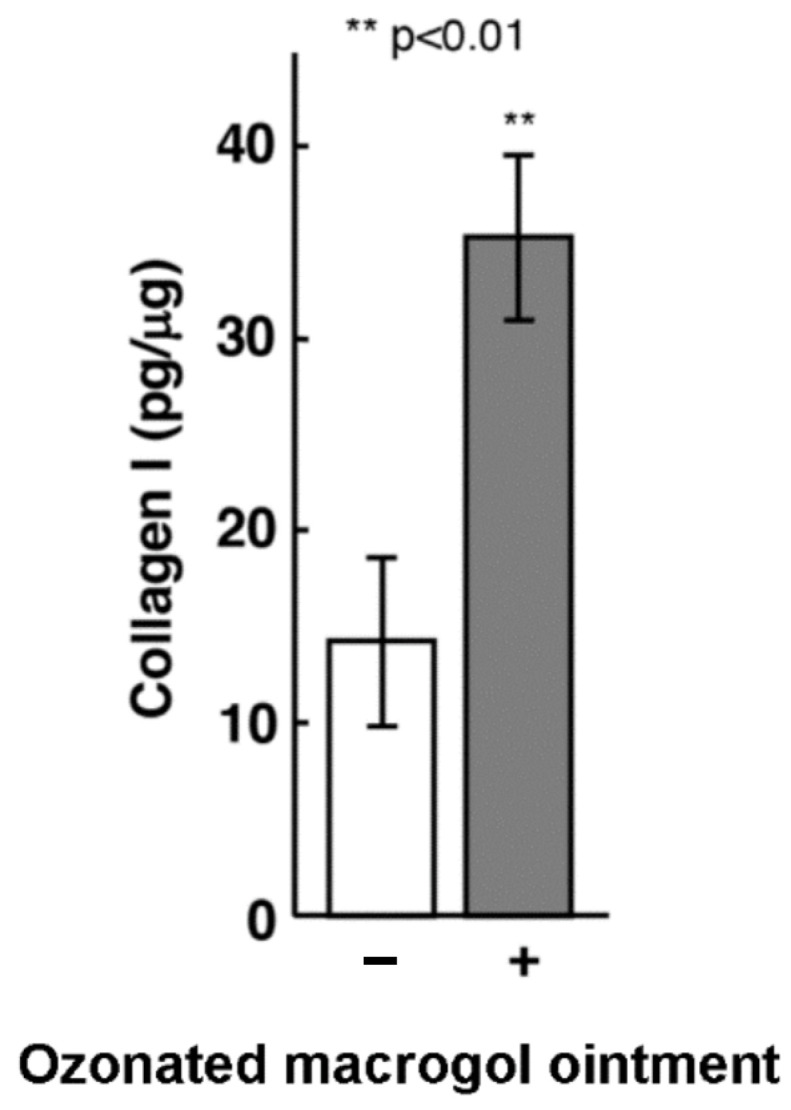
Effects of ozonated macrogol ointment on type 1 collagen production in Saos-2 cells. Saos-2 cells (1 × 10^4^) were seeded into the 96-well plate and exposed to a medium containing 0.5 ppm ozone macrogol ointment for 24 h. Protein levels of type 1 collagen in the media were measured by ELISA. Data are presented as mean ± standard deviation (*n* = 3 in each group). ** *p* < 0.01 vs. control, analyzed using Student’s *t*-test.

**Figure 3:**
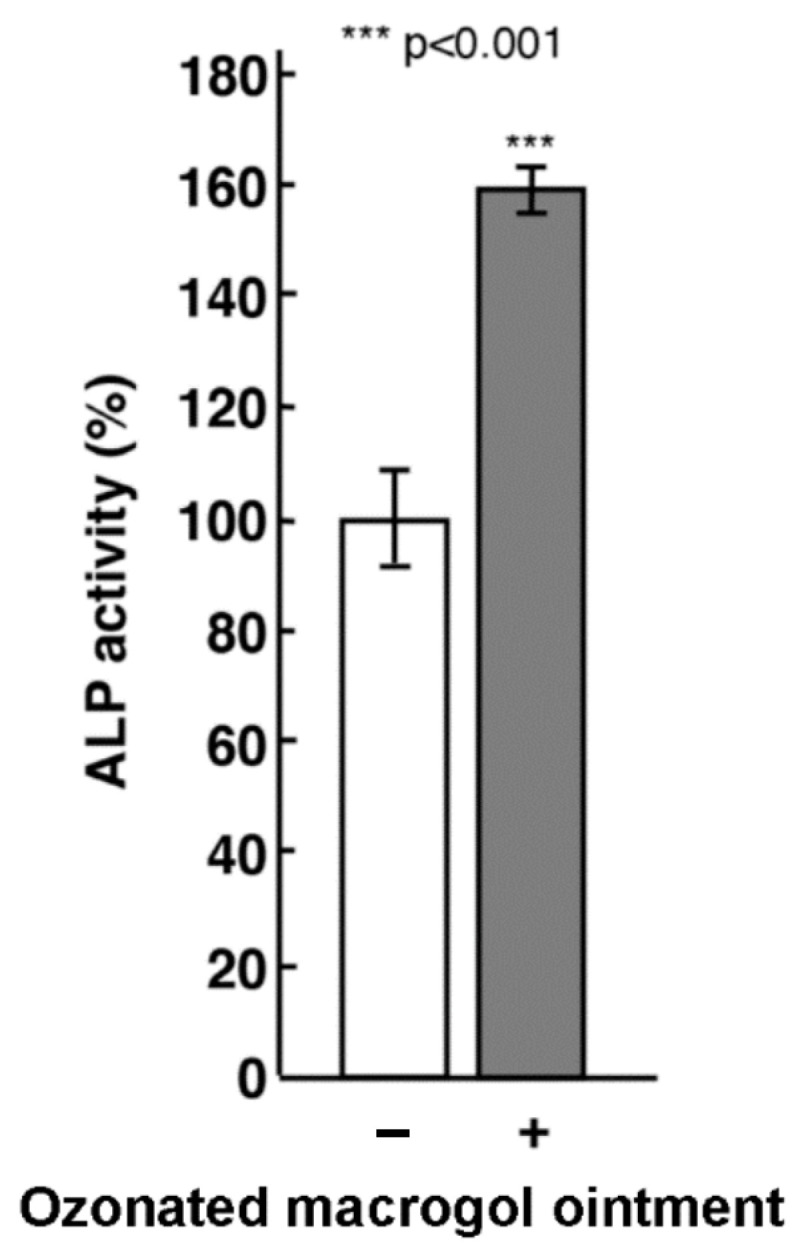
Effects of ozonated macrogol ointment on alkaline phosphatase (ALP) activity in Saos-2 cells. Saos-2 cells were seeded into the 96-well plate and exposed to media containing 0.5 ppm ozonated macrogol ointment for 24 h. Levels of ALP activity in Saos-2 cells were measured using a LabAssay ALP kit. Data are presented as mean ± standard deviation (*n* = 3 in each group). *** *p* < 0.001 vs. control, analyzed using Student’s *t*-test.

Given the results of the proliferation assays, we further evaluated the effect of 0.5 ppm ozonated macrogol ointment on type 1 collagen production and ALP activity by Saos-2 cells. The medium containing ozonated macrogol ointment effectively increased type 1 collagen production (Figure 2) and ALP activity (Figure 3) by Saos-2 cells compared to the control.

## 4. Discussion

Ozonated glycerol has low viscosity and moderate fluidity because its main ingredient is glycerol. On the other hand, ozonated macrogol ointment is an ointment made by mixing ozonated glycerol with PEG 400 and PEG 4000, and has high viscosity and retention at the affected site. The viscosity of the ozonated macrogol ointment was about two times higher than that of the ozonated glycerol. The present study showed that although high concentrations of ozonated glycerol ointment, such as ozonated glycerol [13], also decreased the cell viability index, low concentrations of ozonated glycerol ointment enhanced cell proliferation. Previous reports have suggested that 10 ppm ozone gel suppresses the proliferation of human gingival fibroblasts [12]. In this study, it was demonstrated that while high concentrations of ozonated macrogol ointment (5 ppm) significantly inhibited cell proliferation, concentrations of 0.5 ppm or lower were sufficient to promote cell proliferation. On the basis of these observations, we conclude that ozonated macrogol ointment exhibits pharmacological effects without cytotoxicity at the available low concentrations.

Type 1 collagen and ALP are widely known as markers secreted in the early phases of bone formation [20]. In this study, these initial-phase bone matrix markers were secreted at an optimal ozonated glycerol ointment concentration (0.5 ppm). The effects of ozonated macrogol ointment on type 1 collagen production and ALP activity in Saos-2 cells were also investigated. Our results showed that ozonated macrogol ointment, such as ozonated glycerol, also regulates cell metabolism in osteoblasts, resulting in the secretion of early bone-related biomarkers. However, ALP activity was slightly lower for ozonated macrogol ointment than for ozonated glycerol. ALPs are membrane-bound ectoenzymes that hydrolyze monophosphate esters at a high pH (pH 8–10) [21]. There have been no reports of biocompatible PEG inactivating ALP. In addition, hydroxyapatite-PEG hydrogel composites are used as a scaffold for cell adhesion, which rather enhances the ALP activity of adherent cells [22]. Therefore, PEG may act to delay the arrival of ozone to ALP by gently interacting with the cell membrane around ALP. However, since ozonated macrogol ointment also increases ozonated glycerol ALP activity, this comparative study indicates that ozonated macrogol ointment can be used for clinical purposes, and has similar bone matrix production activity as ozonated glycerol in Table 1. Glycerol and macrogol are ingredients with a high safety profile and proven track record, and their application in wound care and other medical settings could potentially offer clinical benefits. Therefore, these materials could be considered for use in open wounds and other wound types. However, in cases where medical device or drug approval is required, further data may be necessary for regulatory submission, which could be considered a disadvantage. Although this study examined the effects of macrogol addition at the cellular level, it provides a foundation for future investigations into its clinical efficacy. Further research is necessary to evaluate the effects of macrogol addition in clinical settings, and these findings can serve as a starting point for such studies.

In summary, we compared the ozonated glycerol and macrogol ointment. The effect of ozonated macrogol ointment on type 1 collagen production and ALP activity in Saos-2 cells was investigated. The results indicated that ozonated macrogol ointment controls the cellular metabolism of osteoblasts, resulting in the secretion of early bone-related biomarkers as well as ozonated glycerol. Currently, we shed light on the functional studies of ozonated macrogol ointment on long-term effects in terms of cellular damage toward clinical application.

## Figures and Tables

**Table 1:** Comparison of ozonated glycerol and macrogol ointment ^1^.

	Concentration of Ozone (ppm)	Ozonated Glycerol ^1^	Ozonated Macrogol Ointment
MTT assay (%)	0.5	135.0 ± 1.5	156.8 ± 13.1
DNA synthesis (fold)	0.5	2.0 ± 0.04	2.4 ± 0.16
type 1 collagen (pg/μg)	0.5	36.2 ± 3.4	35.3 ± 4.3
ALP (%)	0.5	224.6 ± 15	157.8 ± 5.8

^1^ The data were referred to [13].

## Data Availability

Data sharing is not applicable.
